# Biodiversity and Biological Interactions of Actinobacteria Associated with Deep Sea and Intertidal Marine Invertebrates

**DOI:** 10.3390/md23100408

**Published:** 2025-10-17

**Authors:** Hosea Isanda Masaki, Yannik Karl-Heinz Schneider, Ole Hinnerk Franz, Espen Holst Hansen, Jeanette Hammer Andersen, Teppo Rämä

**Affiliations:** Marbio, Faculty of Bioscience, Fisheries and Economics, UiT-The Arctic University of Norway, 9037 Tromsø, Norway; yannik.k.schneider@uit.no (Y.K.-H.S.); ole.h.franz@uit.no (O.H.F.); espen.hansen@uit.no (E.H.H.); jeanette.andersen@uit.no (J.H.A.); teppo.rama@uit.no (T.R.)

**Keywords:** actinobacteria, marine invertebrates, metabarcoding, high-throughput co-cultivation, antimicrobial activity, bacterial interactions

## Abstract

Studying marine Actinobacteria across ecological niches is essential for discovering novel natural products and understanding microbial interactions. In this study, we investigated the diversity of Actinobacteria associated with five Arctic marine invertebrates using both selective culture-based techniques and culture-independent methods. Additionally, we investigated bacteria–bacteria interactions in an advanced high-throughput co-cultivation assay. We isolated 25 Actinobacteria and classified them into 15 genera, with 53% of the isolates recovered from the sponge *Halichondria panicea*. In contrast, metabarcoding revealed a high diversity of Actinobacteria, with *Chlamys islandica* exhibiting the highest uniqueness of Amplicon Sequence Variants (ASVs), as 21.76% of its ASVs were found exclusively in this species. Similarly, not only did *Dendrobeania* sp. and *Tricellaria ternata* display notable levels of unique ASVs at 19.91% and 18.06%, respectively, they also shared 17.74% of ASVs, demonstrating a greater similarity in their microbial communities than between more distantly related hosts. A variety of microbial interactions were observed on solid medium, including both cooperative and antagonistic relationships, using the co-cultivation assay. These included inter- and intra-Actinobacteria interactions, as well as interactions with human pathogenic bacteria. The duration of co-cultivation and the physical proximity of bacterial partners influenced the extent of these interactions.

## 1. Introduction

Globally, the number of bacterial species is estimated to range from 0.8 to 1.6 million species, with only about 1% formally identified [1]. Identifying new bacterial species enhances our understanding of biodiversity, particularly in less-explored habitats. Actinobacteria represent the most economically and biotechnologically significant phylum of Bacteria, primarily due to their high potential for the discovery of natural products [2,3]. These bacteria display different morphologies and are typically recognized by their cord-like growth mycelium characterized by branched filaments and the ability to produce spores, particularly under harsh environmental conditions [4,5]. However, not all Actinobacteria can sporulate [6]. The genus *Streptomyces* serves as a significant reservoir for pharmaceutical compounds, with approximately 75% of all bioactive secondary metabolites derived from Actinobacteria attributed to this genus [7]. Nevertheless, extensive screening of Actinobacteria isolated from terrestrial environments over several decades has led to the repeated isolation of known producer strains and known compounds, posing a substantial challenge to identifying novel antibiotic agents [8]. Therefore, the focus in biodiscovery has shifted towards Actinobacteria strains isolated from underexplored habitats, such as largely uncharted and extreme marine environments, as these may provide additional potential for the discovery of novel compounds [9,10]. Among the studied marine organisms, invertebrates and particularly sponges (phylum Porifera), are known for their symbiosis with microorganisms and are therefore of particular interest. Culturing Actinobacteria from marine invertebrates is difficult because these bacteria often form close symbiotic relationships with their hosts, grow slower and are outcompeted by faster-growing bacterial cells. Therefore, successfully isolating symbionts heavily depends on the conditions that maintain symbiont–host specific interactions [11]. Consequently, effective cultivation necessitates specialized isolation techniques, and broad cultivation approaches are essential to ensure successful isolation.

Experimental approaches, such as co-cultivation, have been employed to explore the biosynthetic potential of Actinobacteria in producing complex natural products. This approach influences the expression of numerous genes, resulting in significant changes in the regulation of specialized metabolic pathways [12]. Interaction assays using co-cultivation of microbes have proven to be an important method for studying microbe-microbe interactions and the production of natural compounds that may only be produced as a result of the interplay between two or more individual strains [13]. Co-cultivation can be achieved by mixing different liquid cultures and spotting or streaking them in close proximity or intersecting patterns on solid nutrient agar [14]. While the liquid culture approach allows for rapid upscaling and potential mass production of compounds, it is much easier to visually assess the interactions and reactions when observing colonies on solid media. When faced with many isolates to be tested, manual streaking or spotting becomes time-consuming and challenging to replicate consistently. Manually reproducing precise inter-inoculum distances and accurately dispensing single-microliter inocula is both challenging and time-consuming. Automation and robotics can be used to overcome the challenges related to accuracy and low throughput. However, only a few studies have employed robotics as a high-throughput screening approach for Actinobacteria. Notably, studies using AlamarBlue^®^ have demonstrated its utility in providing rapid and highly consistent assessments of both growth-inhibiting and growth-promoting metabolites. This method has been applied to groundwater bacteria, including Actinobacteria, and to investigate how interspecific interactions influence antimicrobial activity in soil bacteria [15,16].

This study primarily aimed to combine culture-dependent and metabarcoding approaches using Illumina, a high-throughput sequencing (HTS) technique, to assess the diversity of Actinobacteria in marine invertebrate samples from the Arctic region of Norway. We hypothesized that isolating Actinobacteria from underexplored habitats and hosts would reveal new taxa with the potential to produce natural products with biological activities. To address the challenges of isolating especially slow-growing Actinobacteria, we used heat shock treatment on the samples to eliminate fast-growing bacteria. Additionally, we sought to explore the biosynthetic potential of cultivable Actinobacteria through in-house developed interaction assays where Actinobacteria were challenged against each other and human pathogenic bacteria using an automated colony picker robot (QPix^®^, Molecular Devices, Sunnyvale, CA, USA) to demonstrate its high-throughput capabilities in co-cultivation experiments.

## 2. Results

### 2.1. Taxonomy and Distribution of Cultured Actinobacteria

**Figure 1:**
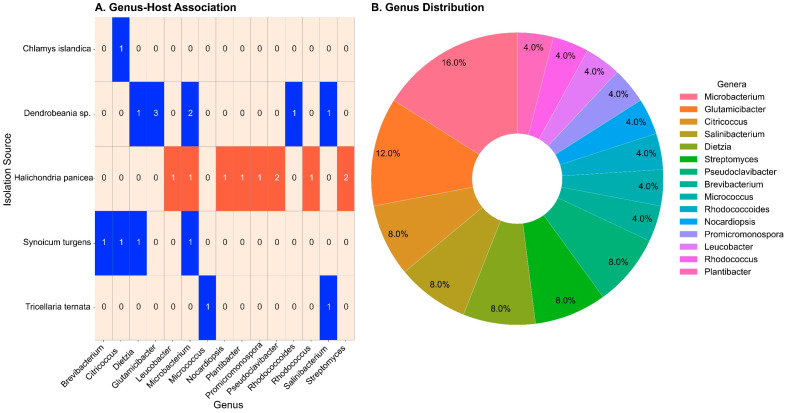
Distribution of Actinobacteria isolates by genus per host and treatment. (**A**) Shows isolate counts for different Actinobacteria genera across the five source invertebrates. Isolates coming from heat shock pre-treatment are highlighted in red. Isolates from the control group, where samples were not heat-treated, are shown in blue. (**B**) Displays the overall relative abundance of Actinobacteria genera across all samples.

Five marine invertebrate samples, *Synoicum turgens* (Chordata), *Dendrobeania* sp. (Bryozoa), *Tricellaria ternata* (Bryozoa), *Chlamys islandica* (Mollusca), and *Halichondria panicea* (Porifera), were used to selectively isolate Actinobacteria using a heat shock method and a single agar medium. During incubation, isolation plates were examined periodically, and colonies displaying different morphologies were picked and streaked in FMAP agar plates to obtain pure cultures. Most of the colonies on the isolation plates became visible after two weeks of incubation. However, some colonies became visible after three months. In total, we successfully recovered 78 isolates. Nearly full-length 16S rRNA gene sequences were generated, and based on BLAST v2.17.0 results for homology and similarity, all identified isolates not belonging to the Actinobacteriota phylum were excluded from this study. Consequently, they were neither considered for further downstream analysis nor preserved for future use. Of the recovered isolates, 25 (32%) belonged to Actinobacteria (Table 1), including 10 from the *H. panicea* host, 4 from *S. turgens*, 8 from *Dendrobeania* sp., 2 from *T. ternata*, and 1 from *C. islandica*. Using BLASTn algorithm on the NCBI database (https://www.ncbi.nlm.nih.gov/ accessed on 21 November 2024) and the Ribosomal Database Project (RDP-II) classifier program [17], isolates were classified into 15 genera (Figure 1): *Brevibacterium*, *Citricoccus*, *Dietzia*, *Glutamicibacter*, *Leucobacter*, *Microbacterium*, *Micrococcus*, *Nocardiopsis*, *Plantibacter*, *Promicromonospora*, *Pseudoclavibacter*, *Rhodococcoides*, *Rhodococcus*, *Salinibacterium*, and *Streptomyces*, representing eight Actinobacteria families (Figure S1). Three Actinobacteria isolates—A03, A032, and HB28—exhibited 16S rRNA gene similarity values of 98.54%, 97.81%, and 98.54%, respectively, to their closest matches in the NCBI database. These values are below the 98.65% threshold commonly used to differentiate between bacterial species [18].

Pre-treatment methods for selectively isolating marine-derived actinomycetes usually use temperatures of 50 °C and 55 °C [19]. To improve the heat-shock strategy for the recovery of Actinobacteria, we employed two different sample pre-heating conditions (55 °C and 65 °C, respectively) alongside a control group, rather than using 55 °C as previously described [20]. Of the total number of isolates (78), 43 were from the control group, which was not subjected to heat treatment, and they included both Gram-negative and Gram-positive bacteria. Based on 16S rRNA sequence BLAST results, 15 isolates (34.9%) were identified as Actinobacteria, while 28 isolates were identified as non-Actinobacteria. The 15 Actinobacteria were isolated from four samples, with no Actinobacteria isolated from *H. panicea* under the control conditions. Using the heat-shock approach, 35 isolates were obtained from the fresh *H. panicea* sample, with none isolated from the frozen samples of *S. turgens*, *Dendrobeania* sp., *T. ternata*, and *C. islandica*. Based on 16S rRNA sequence analysis, 25 were identified as Bacilli and 10 as Actinobacteria. Equal numbers of genera (8 each) were recovered under both heat shock and control conditions, with *Microbacterium* isolated from both (Figure 1). At 55 °C, six isolates were recovered, whereas at 65 °C, four isolates were obtained (Table 1).

### 2.2. Taxonomic Composition of Actinobacteriota Across Metabarcoding Samples

**Figure 2:**
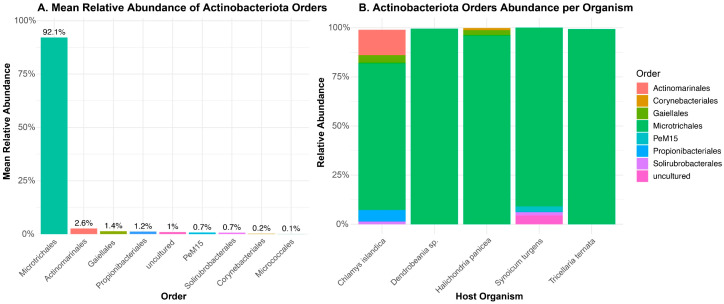
Abundance and community composition of Actinobacteria at the order level and across host organisms. (**A**) The mean relative abundance of Actinobacteriota orders across all samples, (**B**) The relative abundance of Actinobacteriota orders per host organism, illustrating the compositional differences in Actinobacteria communities among invertebrate hosts.

The metabarcoding analysis generated 5470 unique ASVs from the 4,568,671 reads. Our assessment of sequencing depths per sample revealed varying depths, with *Halichondria panicea* displaying the highest depth (Figure S2). The rarefaction curves further confirmed the depth of our sequencing efforts, an indication that the total bacterial community was captured. After normalization and filtering out non-Actinobacteria, we obtained 214 ASVs belonging to Actinobacteria communities associated with the five hosts (Figure S2). To evaluate which taxa were abundant in the Actinobacteria dataset, we calculated the mean relative abundance for each order across all samples. In total, 6 known Actinobacteria orders were identified, with two remaining unclassified, likely due to their rarity. Microtrichales were by far the most dominant taxon, with a mean relative abundance of 92.1%, followed by Actinomarinales at 2.0% (Figure 2). Actinomarinales were predominantly identified in the *Chlamys islandica* host sample, whereas “uncultured” were mostly observed in the *Synoicum* host. Other low-abundance taxa included Solirubrobacterales, Corynebacteriales, and Micrococcales.

### 2.3. Alpha Diversity Analysis of Marine Actinobacteria Communities

The calculated alpha diversity metrics provided insights into the richness and evenness of Actinobacteria communities across the host organisms. The observed ASV richness varied substantially between hosts, with *Dendrobeania* sp. and *T*. *ternata* exhibiting the highest richness, each exceeding 100 unique ASVs (Figure S3). In contrast, *S*. *turgens* and *H*. *panicea* showed markedly lower richness, with fewer than 40 ASVs, underscoring the host-specific variations in Actinobacteria diversity. Shannon diversity, which accounts for richness and evenness, was higher in *T*. *ternata,* followed by *C*. *islandica,* demonstrating the greatest diversity and suggesting a high number of ASVs and a more even distribution. The Simpson diversity, which emphasizes dominance, remained consistently high across all samples (>0.75), indicating that despite differences in richness, the Actinobacteria communities were generally even and not dominated by a single taxon. Notably, all samples exhibited comparable Simpson diversity indices, suggesting a relatively even and balanced Actinobacteria community structure.

### 2.4. Distribution of Shared and Unique Actinobacteria ASVs Among Marine Host Organisms

**Figure 3:**
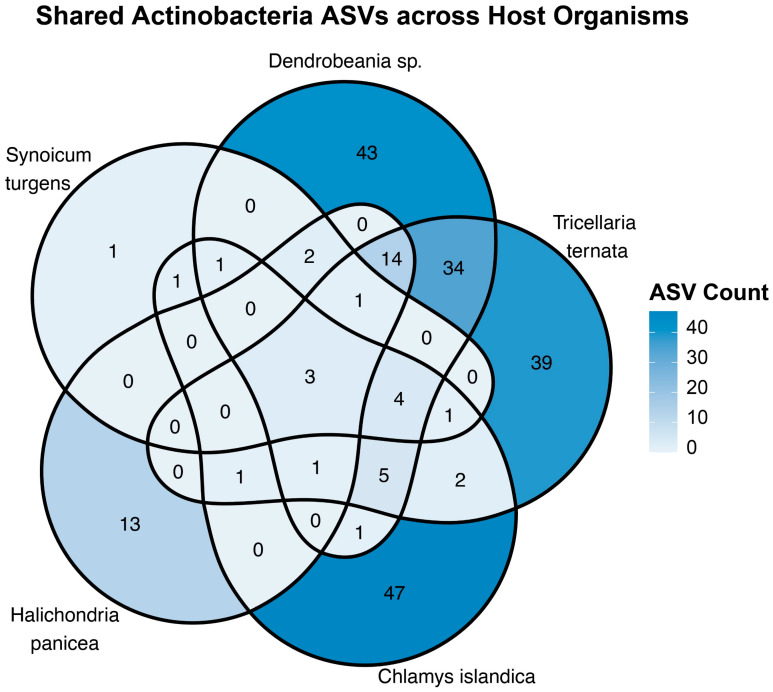
Venn diagram showing the distribution of Actinobacteria ASVs across host organisms. Each area represents the number of ASVs that are unique or shared among hosts, with shading indicating their relative abundance.

To elucidate the distribution of Actinobacteriota ASVs across host organisms, a Venn diagram was constructed (Figure 3). Analysis revealed a high proportion of ASVs that were identified as unique to each host organism, with *C. islandica* exhibiting the highest degree of uniqueness, with 47 ASVs. Similarly, *Dendrobeania* sp. and *T ternata* demonstrated substantial unique ASV counts of 43 and 39, respectively. *S. turgens* had the least unique ASVs (1). Further examination revealed that 34 ASVs were exclusively shared between *Dendrobeania* sp. and *T ternata*. Conversely, only 3 ASVs were identified as shared across all five host organisms. Overall, the analysis of shared ASVs among multiple host combinations revealed that the majority of overlaps consisted of fewer than five ASVs.

### 2.5. Comparative Evaluation of Culture-Dependent and Culture-Independent Methods for Actinobacteria Recovery

**Figure 4:**
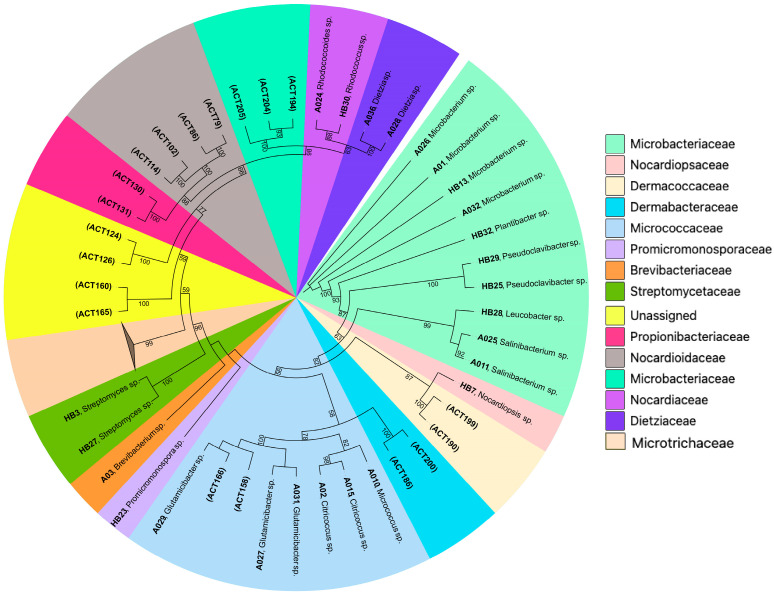
A maximum likelihood phylogenetic tree depicting the relationships between cultured Actinobacteria isolates and ASVs. Cultured isolates are labeled by genus, while branches are color-coded by family to indicate taxonomic affiliation. The clade, composed of 168 ASVs within the family Microtrichaceae, is shown in a collapsed form. Node numbers represent bootstrap support values based on 1000 resamplings, with only values above 50% displayed.

To compare the diversity of Actinobacteria isolates with that of the high-throughput sequencing from the sampled hosts, we compared the 25 isolated Actinobacteria to the 216 representative ASVs assigned to the phylum Actinobacteriota before normalization. Using a ≥97% sequence identity threshold in local BLAST searches, only five ASVs showed close correspondence with the cultured isolates, highlighting a limited overlap between the two approaches (Table S2). Of these, ACT166 was the only ASV with a strong match (>98.9% identity) to three cultured isolates, A027, A029, and A031, all of which were identified as *Glutamicibacter* sp. and derived exclusively from the host *Dendrobeania* sp. The corresponding ACT166 sequence also matched (100%) *Glutamicibacter bergerei* (NR_025612.1) in NCBI’s database, further confirming its taxonomic placement. The remaining 22 isolates showed ≤96% identity to any ASV, revealing that they were not present in the culture-independent dataset. Additional BLASTn searches of unmatched ASVs against NCBI revealed that ACT165 and ACT199 could not be definitively assigned to any known genus within Actinobacteriota; thus, we classified them as rare or underrepresented taxa. In contrast, ACT200 and ACT205 were confidently identified as *Brachybacterium* and *Mycobacterium*, respectively, with greater than 99% identity; however, no cultured isolates corresponded to these ASVs. To assess the phylogenetic relationships between the cultured isolates and the Actinobacteria ASVs, maximum likelihood phylogenetic analysis showed that, although 19 ASVs aligned with clades represented by the 25 cultured Actinobacteria isolates, most grouped into phylogenetically distinct families not found in our cultured collection (Figure 4).

### 2.6. High-Throughput Screening of Culturable Actinobacterial Interactions

**Figure 5:**
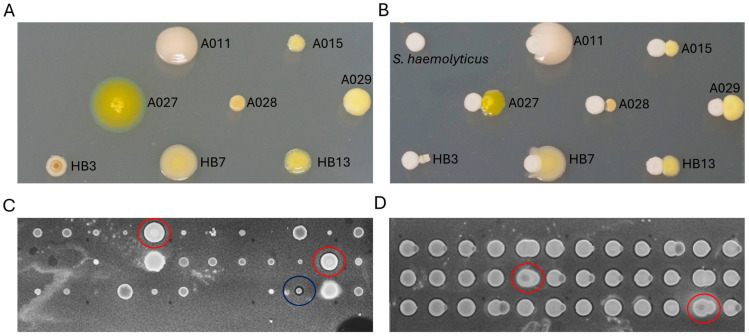
Co-cultivation effect on marine actinobacteria. (**A**,**B**) Images were taken after 96 h of incubation at 25 °C. Size differences between reference (**A**) and *S. haemolyticus* co-cultivation conditions (**B**) can be easily seen (e.g., A027), as well as engulfment (A011 and HB7) and different barriers, ranging from reduced growth (A28) to re-localized structures at the contact zone (A027) and different degrees of separation without overgrowth or mixing (A015, A029 and HB13). Changes in pigmentation and colony topology can be observed (HB3). (**C**) QPix image of a S. aeruginosa agar overlay on the actinobacteria isolates without direct co-cultivation partner. Isolates A011 (red circle) and HB03 (Blue circle) show dark inhibition halos. The dark area in the bottom right represents the halo of a positive control, consisting of a 2 µL drop of 10 mg/mL gentamycin. (**D**) QPix image of *S. aeroguinosa* agar overlay on the isolates (**right**) co-cultivated with the isolate A011 (**left**), showing inhibition halos with the exception of the combination HB7 and A011 (red circle). Details on isolate positions in (**C**,**D**) can be found in Figure 6B.

We developed and implemented a high-throughput screening protocol to study pairwise interactions among phylogenetically diverse, culturable Actinobacteria. Using an automated colony picker for high-throughput and standardized cultivation, we screened co-cultures of Actinobacteria to identify interaction phenotypes characterized by (i) morphological alterations in either or both interacting strains, (ii) competitive inhibition indicative of biological interactions. The experimental matrix included 18 co-cultured Actinobacterial isolates, resulting in 324 unique binary interaction assays (Figure S5). Co-cultures were monitored alongside monoculture controls over a 14-day incubation period, with phenotypic assessments every 24 h. Initial screenings at 24 h post-inoculation revealed minimal interaction due to limited colony growth; however, by 48 h, proximity-dependent interactions became apparent, leading to distinct morphotypic changes between 48 and 96 h (Figure S6). Observed phenotypes included clear (i) zones of inhibition at interfaces, (ii) asymmetric or crescent-shaped colonies indicating directional interference, and (iii) overgrowth of one strain by another where there was complete invasion of one colony by the other (Figure 5A,B). Agar overlay assays with the human pathogen *Streptococcus agalactiae* as an indicator organism showed consistent zones of inhibition linked to isolates HB3 (*Streptomyces* sp.) and A011 (*Salinibacterium* sp.), suggesting that these isolates produced bioactive compounds with potential antibacterial effects (Figure 5C,D). The inhibitory effects were observed in both co- and monoculture conditions, except for the co-culturing with isolate HB7, which suppressed A01’s antimicrobial activity (Figure 5D).

**Figure 6:**
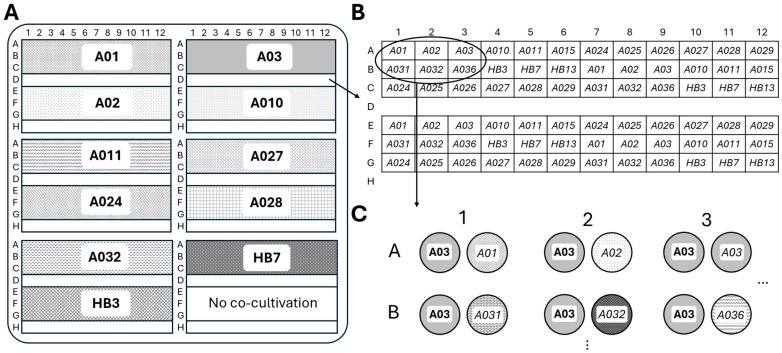
Co-cultivation patterns used in high-throughput assays. (**A**) QTray arrangement of areas co-cultivated with the same isolate. Each gridding of a 96-well plate consisted of two different sections of isolates, separated by an empty row. (**B**) The 96-well plate contained all isolates in replicates, which were gridded across each of the six positions on the QTray. (**C**) A magnified section of the area indicated in B shows the final colony arrangement on the QTray. Isolate names in bold refer to the constant partner in each section outlined in A. Colonies named in italics originate from the isolate plate depicted in B.

## 3. Discussion

### 3.1. Isolation of Culturable Actinobacteria Associated with Marine Invertebrates

In this study, we aimed to investigate the diversity of Actinobacteria in marine invertebrates using both culturing and culture-independent approaches. It is not possible to recommend a single procedure for the selective isolation of diverse Actinobacteria present in environmental samples, given their varied growth requirements. Therefore, we employed and studied the effectiveness of a heat-shock strategy in enhancing the recovery of various Actinobacteria from five marine invertebrate hosts as part of our cultivation-based approach. A total of 25 Actinobacteria isolates (32% of all isolates) belonging to 15 distinct genera were recovered, illustrating the wide taxonomic diversity from a small sample size. All 35 bacterial isolates recovered from the heat-shocked samples were exclusively derived from freshly processed *Halichondria panicea*, with Actinobacteria representing 28.6% of the heat-resistant microbiota. In the control group, bacterial colonies grew from samples of *Synoicum turgens*, *Dendrobeania* sp., *Tricellaria ternata*, and *Chlamys islandica* that had been stored in the freezer, but not from *H. panicea*. Remarkably, all isolates from heat-shocked samples were Gram-positive (either Actinobacteria or Bacilli), indicating that heat shock selectively inhibited the growth of Gram-negative bacteria while promoting the spore-forming Gram-positive taxa. Moreover, the contribution of *H. panicea* to half of the total recovered genera emphasizes the host’s unique potential as a reservoir of culturable Actinobacteria diversity under stressed conditions. It is known that sponges, such as *H. panicea*, comprise up to 40% of their biomass of microbial symbionts, which harbor a vast reservoir of Actinobacteria [21,22,23,24,25]. Both heat pre-treatment and the freshness of the material may have played roles in the cultivability of Actinobacteria, as fresh material was used for isolation from *H. panicea*. In contrast, the other four invertebrate samples were preserved at −80 °C with 20% glycerol for four years from the time of collection until their isolation. However, the low number of culturable Actinobacteria from *S. turgens*, *Dendrobeania* sp., *T. ternata*, and *C. islandica* in this study aligns with the previous study that isolated Actinobacteria from the same samples when they were fresh [20]. They recovered Actinobacteria isolates solely under non-pretreatment conditions, except for one isolate from *S. turgens*. Interestingly, all the genera we recovered from *S. turgens* and *Dendrobeania* sp. were also found in fresh samples from the study by Schneider et al. [20], except that we isolated *Glutamicibacter* from *Dendrobeania* sp. in our study. A literature search on the isolation of Actinobacteria from *S. turgens*, *Dendrobeania* sp., *T. ternata*, and *C. islandica*, aimed at understanding the diversity of culturable Actinobacteria and their isolation techniques, reveals that little to no information is publicly available. Therefore, to effectively recover a higher diversity of cultivable Actinobacteria, integrating both sample pre-treatment and a selective medium will be critical.

Importantly, this study identified three isolates with less than 98.65% 16S rRNA gene sequence similarity to known species, indicating the potential discovery of novel Actinobacteria [18]. Diversity of genera varied between treatments, with *Microbacterium* being the only genus common to both. The heat-shock treatment yielded eight unique genera, and the control treatment also yielded eight unique genera. Despite the number of unique genera being similar, the composition and the ratios of Gram-negative to Gram-positive bacteria differed, suggesting that pretreatment methods can alter recovered community structure without necessarily increasing genus diversity. The selective inhibition of Gram-negative bacteria by heat treatment favored the growth of Gram-positive taxa, including spore-forming Actinobacteria, highlighting the importance of tailored pre-treatment methods.

### 3.2. Actinobacteria Community in the Culture-Independent Approach

Metabarcoding analyses revealed that Actinobacteria communities associated with host organisms formed distinct, host-specific assemblages, highlighting the influence of host identity in shaping their composition. We identified 214 ASVs belonging to the phylum Actinobacteriota, and targeted taxonomic filtering with rarefaction analysis confirmed that the sequencing depth was sufficient for capturing a significant portion of the whole Actinobacteria communities present in the invertebrates. Taxonomic profiling across invertebrate hosts was overwhelmingly dominated by Microtrichales (>90% relative abundance), suggesting a conserved or host-associated role. In a study on sediment samples from Helgoland (North Sea) and Isfjorden (Svalbard), it was found that uncultured Actinomarinales and Microtrichales constituted a major group, making up 8 ± 1% of total reads in Helgoland and 31 ± 6% in Svalbard in the Actinobacteriota phylum [26]. The abundance of this family has also been found to be enriched in bottom water masses, with their relative abundance increasing with increasing sea depth [27]. The low-abundant taxa, such as Micrococcales and Propionibacteriales, were observed particularly in *C. islandica* and *H. panicea*. This dichotomy implies a potential functional partitioning, where core Microtrichales fulfill broad symbiotic roles, while rarer taxa may respond to host-specific ecological or physiological constraints [28].

To understand Actinobacteria community across hosts, metabarcoding data analysis revealed that *Chlamys islandica* had the highest proportion of unique ASVs, with 21.76% found exclusively in this species. This was closely followed by the bryozoans *Dendrobeania* sp. (19.91%) and *Tricellaria ternata* (18.06%). The bryozoans shared the highest proportion of the same ASVs (17.74%), possibly due to their similar complex microhabitats and chemical cues that promote bacterial colonization [29,30,31]. Their sampling from the same station may also explain this, or their evolutionary relatedness, since both belong to Gymnolaemata, one of the three classes of bryozoans [32]. In contrast, the sponge (*Halichondria panicea*) and ascidian (*Synoicum turgens*) exhibited the lowest proportion of unique ASVs (6.07% and 0.47%, respectively), which may reflect stronger host filtering mechanisms or limited ecological niches for bacterial colonization despite marine sponges (*Haliclona* sp., *Callyspongia* sp., and *Desmacella* sp) and tunicate organisms in the class of Ascidiacea being known to harbor rich bacterial communities, including members of the phylum Actinobacteriota [33,34]. Only three ASVs were present in all five hosts, indicating limited overlap in actinobacterial communities among different marine invertebrates. This observation is consistent with previous marine microbiome research, which often highlights the strong connection between microbial communities and both host identity and environmental factors [35,36]. Our findings support the view that the marine environment harbors a high diversity of host-specific bacterial taxa. The host specificity could explain the low diversity of Actinobacteria in *Halichondria panicea* and *Synoicum turgens* [37,38].

### 3.3. Evaluation of Culture-Dependent and Culture-Independent Methods for Actinobacteria Recovery

We investigated the ability of culturing methods to recover the diversity of Actinobacteria as identified by the culture-independent approach. Our findings revealed a clear discrepancy between these two approaches. Out of 25 culturable Actinobacteria, only three *Glutamicibacter* isolates, associated with *Dendrobeania* sp., shared more than 97% sequence identity with ASVs from amplicon sequencing data. The fact that the vast majority of ASVs did not have cultured counterparts suggests that traditional cultivation methods only capture a small fraction of the actinobacterial diversity present in marine invertebrate microbiomes. This bidirectional mismatch highlights the limitations inherent in both approaches. In contrast, although amplicon sequencing is comprehensive, it may underrepresent low-abundance or highly divergent taxa [39] due to primer bias [40], where specific DNA sequences are preferentially amplified over others, the choice of the DNA extraction method, which has been shown to influence the bacterial community profiles generated significantly [41] and database limitations [42]. The exclusive match of *Glutamicibacter* isolates and their corresponding ASVs (ACT166) within a single host organism highlights the potential host specificity of this taxon. The failure to culture dominant ASVs from the Microtrichales order, despite their high sequence identity to database references, further indicates that many ecologically relevant taxa remain inaccessible using standard media and conditions. The dominance of the class Acidimicrobiia, particularly members of the order Microtrichales, in the culture-independent dataset, but not in the culture-dependent one, is consistent with patterns reported in previous studies. A prior study [43] recovered 169 actinobacterial strains from saline environments using culture-based methods, all of which belonged to the order Actinomycetales. In contrast, their culture-independent 16S rRNA gene clone library (*n* = 748) revealed a broader taxonomic diversity, including Actinomycetales, Acidimicrobiales, and several unclassified Actinobacteria. These findings reflect the well-documented discrepancy between cultured and uncultured microbial diversity in environmental microbiology, known as the Great Plate Count Anomaly [44]. Whereas abiotic factors such as organic matter, pH, and nutrients have been shown to influence the composition and structure of Actinobacteria communities [36,45,46], their effect is limited [47] while biotic interactions play a more significant role [48]. Our findings highlight the necessity for more extensive and tailored culturing techniques, such as co-culturing and microfluidic isolation methods, to more accurately represent the in situ diversity shown by sequencing.

### 3.4. Morphological Responses of Culturable Actinobacteria to Co-Cultivation

Microbes form complex communities in which interspecies interactions drive phenotypic and genotypic changes that enhance survival and structure the community within their microenvironment [15,49,50]. By leveraging these naturally occurring interactions, microbial co-culturing has become a promising strategy to activate otherwise cryptic metabolic pathways [12,51,52,53]. Due to inefficiency and reproducibility challenges presented by manual co-culturing, a high-throughput method using robotic systems is essential for screening dozens to hundreds of isolates. This study extends this approach by employing a QPix colony picker robotic platform for high-throughput co-cultivation of Actinobacteria isolated in this investigation, aiming to identify phenotypic interactions that develop over time. Previous studies using the QPix system have typically combined bacterial isolates in liquid culture before spotting them onto solid agar, or spotting co-cultured isolates in identical locations, primarily to stimulate the production of secondary metabolites [15,16]. While effective for chemical induction, this approach does not enable direct observation of morphological interactions, such as distinctive colony growth patterns between partners. In contrast, our study focused on growth directly on solid media by spotting isolates in close proximity at defined distances, allowing us to monitor and capture these interaction patterns over time. Early observations revealed minimal interactions within the first 24 h, suggesting that individual colonies had not yet grown sufficiently to expose inter-strain responses; thus, the interaction range within which individual bacterial cells can interact with one another is limited [54]. After 48 h, various interaction phenotypes emerged, including overgrowth, boundary formation, pigment changes, and growth suppression, all of which indicated competitive or neutral interactions. These findings not only highlight the complex and time-dependent nature of microbial interactions, which may only become apparent after prolonged contact or through quorum-dependent signaling [55], but also their possible ecological roles in shaping microbial communities in host-associated environments.

The reduced colony size of A027 (*Glutamicibacter* sp.) when co-cultured with *Staphylococcus haemolyticus* may indicate subtle metabolic signaling or competition for nutrients. The establishment of distinct physical boundaries and uneven overgrowth patterns in isolates such as A015 (*Citricoccus* sp.), A029 (*Glutamicibacter* sp.), and HB13 (*Microbacterium* sp.) suggest the presence of mutual antagonism or self-recognition systems that prevent intermixing [56]. Furthermore, visible shifts in pigmentation and colony morphology, as observed in HB3 (*Streptomyces* sp.), indicate stress responses or the activation of secondary metabolic pathways triggered by interspecies interactions. Instead of using the supernatant of the co-cultured bacteria isolated to overlay on the selected human pathogens to test for their antimicrobial activity as previously reported [15,16], we overlayed the human pathogen *S. agalactiae* cultures on the co-cultivated Actinobacteria isolates. The *S. agalactiae* overlay assays further highlighted the antimicrobial capabilities of specific isolates. For example, A011 (*Salinibacterium* sp.) and HB3 (*Streptomyces* sp.) displayed zones of inhibition. These inhibitory effects are likely mediated by the production of polyketides, non-ribosomal peptides, or other antimicrobial compounds typical of Actinobacteria, especially genera such as *Streptomyces, Micromonospora*, and *Glutamicibacter* [8]. Strain A011 exhibited antimicrobial activity against *Streptococcus agalactiae* both in monoculture and during co-cultivation with other Actinobacteria, suggesting that A011 is the producer of the active compound. Interestingly, this antimicrobial activity was lost when A011 was co-cultivated with strain HB7. This lack of inhibition in co-culture indicates a suppressive effect of HB7 on A011’s bioactivity, possibly through competitive quorum-sensing interference, downregulation of biosynthetic pathways, or a resource-based metabolic shift, a phenomenon well-documented in co-cultivation models [57,58]. These findings indicate that actinobacterial isolates employ diverse interaction strategies, including competitive exclusion, physical dominance, and antagonism mediated by secondary metabolites. In the Tara Oceans project, it was demonstrated that biotic rather than environmental interactions primarily drive bacterial community structure, with most of these interactions being positive rather than negative [48]. Notably, the high-throughput interaction assays that we applied using the QPix robotic systems serve as screening tools for biotechnologically important traits, such as antimicrobial production, supporting the growing interest in co-cultivation approaches to activate silent biosynthetic gene clusters [12,51].

A key methodological application in this study was the use of the QPix robotic platform, an advanced tool that enables automated, high-throughput, and spatially controlled inoculation of isolates within specific co-cultivation patterns. Our high-throughput screening technique facilitated the design and generation of 324 co-cultivation combinations. In contrast to manual streaking or spot inoculation techniques, which are often labor-intensive, less precise, and not easily reproducible, this high-throughput approach significantly enhances scalability and creates multiple co-cultivation matrices for exploring microbial interactions. Automation of the technique reduces human error and increases the reliability and reproducibility of the results. Our approach for screening the co-cultures revealed multiple interesting interactions that we are currently investigating in detail.

## 4. Materials and Methods

### 4.1. Sample Collection and Processing

A total of five marine invertebrate samples were studied in this research, four of which (*Synoicum turgens, Dendrobeania* sp., *Tricellaria ternata,* and *Chlamys islandica*) were collected by bottom trawling from the seabed using a beam trawl during a research cruise with the Norwegian research vessel Kronprins Haakon in the Arctic Ocean in 2020 (Table 2). A previous study by Schneider and co-workers [20] homogenized these invertebrate samples and stored them at −80 °C with 20% (*v*/*v*) glycerol. The fifth sample was a sponge (*Halichondria panicea*) collected from the lower intertidal zone in Tromsø, Norway (Norwegian Sea). The sponge sample was transported to the laboratory in a cooler box. It was processed fresh by rinsing it thoroughly three times with sterile artificial seawater to remove loosely attached sediment particles and microorganisms. Approximately 200 mg of the sponge in duplicate was homogenized in a sterile 2 mL Eppendorf tube using a sterile disposable plastic pestle, and 1 mL of sterile artificial seawater was added to the homogenate. After this, the sponge sample was immediately used for cultivation and extraction of total environmental DNA (eDNA). The remaining material was then preserved in 20% glycerol at −80 °C for future use.

### 4.2. Media for Isolation and Subculturing

In this study, we used M1 medium to isolate Actinobacteria from four samples preserved in glycerol at −80 °C, as well as from a fresh sample of *H. panicea.* FMAP medium was used for subculturing and further purification of colonies. All media compositions were prepared in 1 L of artificial seawater. The M1 comprised 10.0 g soluble starch (VWR Life Sciences, Leuven, Belgium), 4.0 g yeast extract (Sigma Aldrich, Saint-Quentin-Fallavier, France), 2.0 g peptone (Sigma Aldrich, France, Instant Ocean Sea Salts (Aquarium Systems, Sarrebourg, France), and 18 g agar (Sigma Aldrich, Madrid, Spain). FMAP comprised 5 g peptone, 15 g Difco marine broth (Difco Laboratories, NJ, USA), 20 g Instant Ocean Sea Salts, and 15 g agar. All media were autoclaved at 121 °C for 30 min. The M1 isolation media was allowed to cool to approximately 50 °C before being supplemented with 50 mg/L cycloheximide in ethanol to inhibit fungal growth and 25 mg/L nalidixic acid in 0.3 M NaOH to inhibit the growth of Gram-negative bacteria. 25 mL of each respective medium was poured into individual sterile Petri dishes. For inoculating both Actinobacteria and human pathogens in the high-throughput screening of bacteria–bacteria interactions using the QPix technique, International Streptomyces Project Medium 2 (ISP2) [59,60] media was prepared in 1 L of artificial seawater, consisting of 4.0 g glucose (Sigma Aldrich, St. Louis, MO, USA), 4.0 g yeast extract, 10.0 g malt extract (Sigma Aldrich, France), 9.9 g Instant Ocean Sea Salts and 0.2% (*v*/*v*) trace element solution prepared in 10% MgSO_4_ × 7H_2_O, 0.01% FeSO_4_ × 7H_2_O, 0.01% ZnSO_4_ × 7H_2_O, 0.01% CuSO_4_ × 5H_2_O, 0.01% CoCl_2_ × 6H_2_O all (*w*/*v*) and dissolved in 1 L Milli-Q H_2_O. ISP2 solid media included 2% (*w*/*v*) agar.

### 4.3. Sample Pre-Treatment and Actinobacteria Isolation Strategy

Pre-treatment methods for selectively isolating marine-derived actinomycetes usually use temperatures of 50 °C and 55 °C [19]. In our study, we used a temperature of 55 °C along with an additional 65 °C to evaluate whether this affects the cultivability of Actinobacteria. Two 0.1 mL sample aliquots were transferred from the homogenized invertebrate samples (four stored frozen, one fresh) into a 1.5 mL sterile Eppendorf tube. One aliquot was heated to 55 °C and the other to 65 °C using a heating block. After 10 min, the tubes were immediately transferred to ice for 10 min of cooling before being streaked on M1 plates in a sterile bench. As a control, 0.1 mL of each sample was spread without pre-treatment. Plated Petri dishes were incubated at room temperature in a dark environment. Plates were regularly monitored for colony growth for up to three months, and colonies that exhibited different morphologies were picked and subcultured on FMAP media for further purification.

### 4.4. DNA Extraction, 16S rRNA Gene Amplification, and Sequencing of Culturable Actinobacteria

To extract DNA of all culturable Actinobacteria, single colonies of pure isolates were cultured in 10 mL of FMAP broth in individual 25 mL Erlenmeyer flasks and incubated at room temperature for seven days with shaking at 140 rpm. A volume of 750 µL from each culture was added to an equal amount of 40% sterile glycerol stock, giving a final concentration of 20% in sterile 2 mL cryo-tubes for cryopreservation in a −80 °C freezer. Bacterial cells from the culture were obtained by centrifuging 1 mL of the remaining culture at 14,000 rpm in 1.5 mL Eppendorf tubes (VWR, PA, USA). Cell pellets were then resuspended in 200 µL of sterile Milli-Q water, and genomic DNA extraction was performed using GenElute™ Bacterial Genomic DNA Kits (Sigma-Aldrich, St. Louis, MO, USA) following the manufacturer’s instructions except that DNA was eluted with 60 µL of the elution buffer. DNA concentrations were quantified using the Qubit™ Fluorometer (Invitrogen, MA, USA) and the NanoVue™ Plus spectrophotometer (Healthcare Bioscience, Uppsala, Sweden). A nearly full-length 16S rRNA gene was amplified from the genomic DNA in a polymerase chain reaction (PCR) using the commercial primers 27F (5′-AGAGTTTGATCMTGGCTCAG) as the forward and 1429R (5′-TACCTTGTTACGACTT) as the reverse primers [61]. The PCR mixture (25 µL) included 25 ng/µL DNA (1 µL), 10 µM primers (0.5 µL), 2× DreamTaq Buffer (12.5 µL), and molecular biology-grade water (10.5 µL). The PCR thermocycling conditions comprised 95 °C for 5 min, followed by 35 cycles of 95 °C for 30 s, 55 °C for 30 s, and 72 °C for 1 min with a final extension at 72 °C for 10 min. A 5 µL sample of the PCR amplicons was examined on a 1% (*w*/*v*) agarose gel stained with Gel-Red^®^ Nucleic Acid Gel Stain (Biotium, Fremont, CA, USA) in a 1× TBE buffer (Omega Biotek, Norcross, GA, USA) and visualized on a gel bioimaging system (Syngene, Cambridge, UK). The remaining amount was purified using the QIAquick PCR Purification Kit (Qiagen Inc., Hilden, Germany). The resulting purified PCR product was diluted five times and used for sequencing the forward and reverse strands of the 16S gene. Both the forward and reverse sequencing PCRs had a total volume of 10 µL, containing 0.5 µL of the PCR amplicon, 1 µL of either 27F or 1492R primer (1 µM), 1 µL of BigDye 3.1 (Applied Biosystems, Vilnius, Lithuania) 1 µL of 5× sequencing buffer, and 5.5 µL of autoclaved Milli-Q water performed under the following cycling conditions: 95 °C for 1 min, 35 cycles of 95 °C for 5 s, 47 °C for 30 s, and 60 °C for 4 min. PCR products were sequenced at the University Hospital of North Norway (Tromsø, Norway) using an Applied Biosystems 3130xl Genetic Analyser (Life Technologies/Applied Biosystems, Waltham, MA, USA).

### 4.5. Phylogenetic Analysis and Taxonomic Identification of Actinobacteria Cultures

Raw Sanger sequence reads were assembled, analyzed, and manually edited using Geneious Prime^®^ v2023.2.1 [62] to generate consensus gene sequences. Subsequently, consensus sequences were queried against the 16S ribosomal RNA (Bacteria and Archaea) and the nucleotide collection (nr/nt) databases using the BLASTn algorithm [63] to identify closely related strains in the National Center for Biotechnology Information (NCBI) database. The taxonomic classification of the 16S rRNA sequences from the isolates was obtained using the NCBI BLAST database and Ribosomal Database Project (RDP-II) (Cole et al., 2007) [17] classifier program as a package in R V4.2.0 up to the genus level. BLAST hits for the closely related strains and sequences of the strains previously isolated from *Synoicum turgens*, *Dendrobeania* sp., *Tricellaria ternata*, and *Chlamys islandica* [20] were downloaded and imported into Geneious software v2023.2.1, along with the 25 culturable Actinobacteria. Multiple sequence alignment was then performed using the MAFFT v7.490 plugin [64], and the alignment was manually inspected and adjusted to ensure all sequences had the same starting and ending points. Phylogenetic analysis using Maximum Likelihood (ML) was conducted with IQ-TREE v2.4.0 [65] to infer the evolutionary relationships under these parameters: The General Time Reversible (GTR) substitution model, accompanied by Gamma-distributed rate heterogeneity (GTR + G), was selected as the evolutionary model. To evaluate branch support, 1000 ultrafast bootstrap replicates were used [66] and 1000 SH-like approximate likelihood ratio test (SH-aLRT) [67]. *Bacillus subtilis* strain IAM 12118 (NR_112116.2) was chosen as the outgroup for tree rooting. The final tree was visualized and annotated using Interactive Tree of Life (iTOL) v6 [68]. Bootstrap support values and SH-aLRT values were mapped onto the tree nodes, and the tree was tailored for optimal clarity and presentation.

### 4.6. Environmental DNA (eDNA) Extraction and Amplification of the V4/V5 Region of the 16S rRNA Gene for Metabarcoding

Environmental DNA extraction for 16S rRNA gene metabarcoding was performed from the four invertebrate homogenates stored frozen and one fresh sponge homogenate using the DNeasy PowerSoil Pro Kit (Qiagen, Hilden, Germany). 400 μL of the supernatant from a thoroughly mixed and homogenized sample was transferred into 2 mL tubes containing the beads. The Precellys^®^ 24 lysis and homogenisation machine (Bertin Technologies, Montigny, France) was used for sample bead beating at a maximum speed of 6500 rpm for 30 s. The samples were then cooled on ice for 1 min, followed by a second round of bead beating at the same speed for another 30 s. Subsequent steps were undertaken according to the manufacturer’s instructions on a sterilized laboratory bench. Sterile Milli-Q water served as a negative control to check for potential contamination from either the Milli-Q water itself, the air, or laboratory consumables during the extraction process. A Qubit™ Fluorometer (Invitrogen, USA) was used to quantify the DNA concentration per sample. The hypervariable V4–V5 regions of the 16S rRNA gene were targeted for sequencing. This region was amplified by PCR using the universal primers 515YF (5′-GTGYCAGCMGCCGCGGTAA-3′) and 926R (5′-CCGYCAATTYMTTTRAGTTT-3′) [69]. The repliQa HiFi ToughMix kit (Quanta Bio, Beverly, MA, USA) containing the polymerase enzyme was used in the PCR amplification. The cycling conditions included a 30 s heating step at 98 °C, followed by 35 cycles of 98 °C for 15 s, 58 °C for 15 s, 68 °C for 1 min, and a final extension at 68 °C for 2 min. Five microliters were used to check for amplification on a 1% agarose gel. Extractions were performed on a sterile laboratory bench. To track for possible contaminations, sterile artificial seawater from the batch that was applied for sample homogenization was used as a negative control during extraction. After running the gel with no band in the negative control, the sample was excluded from the final sequencing. Duplicate reactions with PCR amplicons were pooled and purified using the QIAquick PCR Purification Kit. DNA concentrations were quantified using the Qubit™ Fluorometer and the NanoVue™ Plus spectrophotometer. Pooled samples were then sent to the Norwegian Sequencing Centre (Oslo, Norway) for sequencing on an Illumina MiSeq V3 platform, generating 2 × 300 bp overlapping paired-end reads.

### 4.7. Bioinformatics and Statistical Analysis of Metabarcoding Data

Demultiplexed raw paired-end Illumina sequencing reads were processed with Trimmomatic v0.39 [70] to remove low-quality bases and adapters, using a 4-base sliding window approach with a PHRED score cutoff of 15. Reads shorter than 250 bp after trimming were discarded. Quality control was performed using FastQC v0.12.1. The “Adapter Content” module indicated the absence of Illumina adapter sequences in all samples; therefore, no additional adapter trimming was necessary. Results were compiled across all samples using MultiQC v1.14 [71] to create a unified quality report for inspection. Reads were imported into QIIME2 software v2023.9 [72] and processed using the built-in DADA2 plugin pipeline [73]. In DADA2, paired-end reads were merged, sequences were denoised and dereplicated, and chimeras were removed to generate Amplicon Sequence Variants (ASVs). Forward and reverse reads were truncated at 250 bp and 200 bp, respectively, to retain high-quality sequence data while ensuring sufficient overlap for paired-end merging. Sequences shorter than the threshold were discarded. Taxonomic classification of ASVs was performed using the VSEARCH-based consensus taxonomy classifier [74], implemented in QIIME 2. The classifier aligned ASVs against the SILVA 138.1 (release 2022.2) reference database [75], using the full-length, unclustered sequences. VSEARCH performed a global sequence alignment and assigned taxa based on the top-matching reference sequences, applying a consensus threshold to ensure classification confidence. Analyses were conducted using resources provided by Sigma2—the National Infrastructure for High-Performance Computing and Data Storage in Norway. The feature table, taxonomy table, representative sequences, and sample metadata were imported into R and merged. All ASVs not belonging to the Bacteria domain were removed and excluded from further analysis. Rarefaction curves were plotted to visualize the sequencing depth of each sample. Data were normalized by subsampling to the minimum library size using the vegan package v2.6-4 [76]. The Phyloseq package v1.44.0 [77] was used to create a phyloseq object that integrated all merged data files for comprehensive ecological analyses. The data were then filtered to focus solely on the phylum Actinobacteriota. Alpha diversity metrics, specifically the observed ASVs, Shannon index, and Simpson index, were calculated to assess microbial richness and evenness within the samples. To evaluate shared and unique Actinobacteria ASVs across samples, a Venn diagram was constructed using the Venndiagram package [78]. All visualizations were created using the ggplot2 package accessed via the tidyverse framework [79]. Representative ASV sequences, along with the isolate sequences, were aligned using MAFFT [64], and highly variable regions were masked to reduce noise in the phylogenetic analysis. A phylogenetic tree was constructed using the maximum likelihood approach implemented in IQ-TREE [80]. The resulting tree was subsequently visualized and annotated using iTOL.

### 4.8. Comparing Culture-Dependent and Culture-Independent Methods for Recovering Actinobacteria

To evaluate the representativeness of the culture-dependent approach, ASVs assigned to the phylum Actinobacteriota were extracted before data normalization to retain full taxonomic diversity. These ASV sequences were used to construct a local BLAST database in Geneious. The 16S rRNA gene sequences of cultured isolates were then queried against this database to determine sequence-level correspondence. A threshold of ≥97% sequence identity was applied to define a match between isolate sequences and ASVs. To contextualize cultured isolates within the broader actinobacterial diversity, a maximum likelihood phylogenetic tree was constructed, based on the alignment of the isolate and ASV sequences.

### 4.9. High-Throughput Screening of Actinobacteria Interactions in Culture Using Automated Colony Handling

All culturable isolates were screened in interaction assays, including pairwise testing among themselves and against the human pathogens *Streptococcus agalactiae* (type B) and methicillin-resistant *Staphylococcus haemolyticus.* This was accomplished through high-throughput co-cultivation using a QPix 460 (Molecular Devices, CA, USA), equipped with the 96-pin gridding head model X4228 and 96-well microtiter plates (Nunc Catalog number 167008) as the source, while QTrays (Molecular Devices, CA, USA) served as receptacles. QTrays are large (500 cm^2^), rectangular cultivation dishes that hold griddings from six standard microtiter plates. Additionally, an offset setting within the QPix software v.1.6.10 enabled variations in the gridding pattern, allowing more plates to be gridded and facilitating co-cultivations closer to partners than the standard well distance. Actinobacteria cultures were inoculated in ISP2 broth for four days at room temperature, with shaking set to 140 rpm. *Staphylococcus haemolyticus* was inoculated from a −80 °C glycerol stock in 10 mL Mueller-Hinton broth (BD Difco, NJ, USA), and *Streptococcus agalactiae* was inoculated in 10 mL Brain Heart Infusion (BHI) Broth (Merck, Darmstadt, Germany) at 37 °C with shaking incubation at 160 RPM, overnight. Right before the QPix gridding process, 240 µL of these cultures were used to fill the wells of a microtiter plate. QTrays were filled with ISP2 agar (2% *w*/*v*) as the growth medium. On each QTray, one reference area was designated for growing each tested isolate without an immediate co-cultivation partner. The chosen co-cultivation pattern enabled 11 blocks of different co-cultivation partners with one reference location (Figure 6A) and a repeating pattern of all isolates gridded at an offset of 2 mm per each QTray (Figure 6B,C). The three rows of a microtiter plate with 36 wells could accommodate the selected 18 Actinobacteria in two replicates (Figure 6B). After each 3-row set of co-cultivation partners, one row was left empty, creating two distinct sets of partners with all 18 tested Actinobacteria in two replicates per microtiter plate (Figure 6A,B). Incubation of the QTrays was performed at 25 °C after gridding. In addition to the co-cultivation of colonies, a sloppy bacterial agar overlay was performed to detect possible zones of inhibition suggesting production of antibacterial compounds. For this, 0.45% Agar in BHI medium was prepared and cooled to approximately 40 °C. A 5 mL overnight culture of *Str. agalactiae* in BHI medium was added to 100 mL of BHI agar, mixed thoroughly with a magnetic stirrer, and poured immediately onto the QTrays until the entire surface was covered. Incubation of the bacterial agar overlay was conducted at 25 °C, as this temperature was deemed sufficient for the growth of the pathogens. Imaging of the QTrays was performed using the imaging function of the QPix within the picking program, utilizing trans-illumination and the integrated monochrome camera, as well as a consumer-grade digital camera for color photos.

## 5. Conclusions

Our study established a broad taxonomic diversity of Actinobacteria from a small sample of marine invertebrates. We isolated 25 Actinobacteria strains belonging to 15 genera. Applying targeted pre-treatments, such as heat shock, to hosts like *H. panicea* can improve the cultivability of marine Actinobacteria. While the culture-based method captures only a small portion of the overall diversity in our samples, it is an important step toward discovering new bacteria with potential applications in biotechnology. To fully explore the extensive diversity of Actinobacteria and deepen our understanding of microbial life in marine ecosystems, extensive sampling and culturing are essential. This research not only broadens our knowledge of marine microbial communities but also helps guide future scientific studies.

Using a high-throughput sequencing approach, our results demonstrate the presence of a broad and diverse Actinobacteriota phylum in marine invertebrates, primarily dominated by Microtrichales, and indicate the presence of conserved or host-specific functions. Only a few of the Actinobacteria ASVs were shared between individual hosts, with the only exception being invertebrates sampled at the same study site and being phylogenetically closely related.

The QPix robotic platform enabled effective and reproducible screening of co-cultivations, and this approach may be used to prioritize interacting microbial isolates for in-depth ecological, physiological, or natural product discovery studies.

## Figures and Tables

**Table 1:** A summary of the 25 culturable Actinobacteria isolates obtained in this study, including their source of isolation and data from NCBI based on 16S rRNA gene sequence percentage identity. The treatment column indicates whether the samples were subjected to heat treatment at a specific temperature or served as controls where no heat treatment was applied.

Isolate Id	Accession	Phylogenetic Group	Treatment	Host Source	Most Closely Related Taxon (Accession No.)	Identity (%)
A01	PQ814116	Microbacteriaceae	Control	*S. turgens*	*Microbacterium oxydans* (NR_044931)	99.03
A02	PQ814117	Micrococcaceae	Control	*S. turgens*	*Citricoccus parietis* (NR_104498)	98.85
A03	PQ814118	Brevibacteriaceae	Control	*S. turgens*	*Brevibacterium aurantiacum* (NR_044854)	98.54
A010	PQ814119	Micrococcaceae	Control	*T. ternata*	*Micrococcus yunnanensis* (NR_116578)	99.85
A011	PQ814120	Microbacteriaceae	Control	*T. ternata*	*Salinibacterium amurskyense* (NR_041932)	99.64
A015	PQ814121	Micrococcaceae	Control	*C. islandica*	*Citricoccus parietis* (NR_104498)	99.06
A024	PQ814122	Nocardiaceae	Control	*Dendrobeania* sp.	*Rhodococcoides fascians* (NR_037021)	98.83
A025	PQ814123	Microbacteriaceae	Control	*Dendrobeania* sp.	*Salinibacterium amurskyense* (NR_041932)	99.78
A026	PQ814124	Microbacteriaceae	Control	*Dendrobeania* sp.	*Microbacterium oxydans* (NR_044931)	99.96
A027	PQ814125	Micrococcaceae	Control	*Dendrobeania* sp.	*Glutamicibacter bergerei* (NR_025612)	99.35
A028	PQ814126	Dietziaceae	Control	*Dendrobeania* sp.	*Dietzia kunjamensis* (NR_116684)	99.86
A029	PQ814127	Micrococcaceae	Control	*Dendrobeania* sp.	*Glutamicibacter bergerei* (NR_025612)	99.93
A031	PQ814128	Micrococcaceae	Control	*Dendrobeania* sp.	*Glutamicibacter bergerei* (NR_025612)	99.86
A032	PQ814129	Microbacteriaceae	Control	*Dendrobeania* sp.	*Microbacterium invictum* (NR_042708)	97.81
A036	PQ814130	Dietziaceae	Control	*Synoicum turgens*	*Dietzia kunjamensis* (NR_117962)	99.55
HB3	PQ814131	Streptomycetaceae	Heat shock 55 °C	*H. panicea*	*Streptomyces argenteolus* (NR_112300)	99.17
HB7	PQ814132	Nocardiopsaceae	Heat shock 55 °C	*H. panicea*	*Nocardiopsis prasina* (NR_044906)	99.64
HB13	PQ814133	Microbacteriaceae	Heat shock 65 °C	*H. panicea*	*Microbacterium oxydans* (NR_044931)	99.85
HB23	PQ814134	Promicromonosporaceae	Heat shock 65 °C	*H. panicea*	*Promicromonospora iranensis* (NR_109446)	99.75
HB25	PQ814135	Microbacteriaceae	Heat shock 55 °C	*H. panicea*	*Pseudoclavibacter terrae* (NR_145621)	99.92
HB27	PQ814136	Streptomycetaceae	Heat shock 55 °C	*H. panicea*	*Streptomyces niveus* (NR_115784)	98.82
HB28	PQ814137	Microbacteriaceae	Heat shock 55 °C	*H. panicea*	*Leucobacter viscericola* (NR_180647)	98.54
HB29	PQ814138	Microbacteriaceae	Heat shock 65 °C	*H. panicea*	*Pseudoclavibacter terrae* (NR_145621)	99.93
HB30	PQ814139	Nocardiaceae	Heat shock 65 °C	*H. panicea*	*Rhodococcus qingshengii* (NR_043535)	99.92
HB32	PQ814140	Microbacteriaceae	Heat shock 55 °C	*H. panicea*	*Plantibacter flavus strain* (NR_025462)	99.78

**Table 2:** A description of the invertebrate host samples used in this study.

Sample Source	Phylum	Latitude	Longitude	Depth/Intertidal Zone
*Synoicum turgens* (A16)	Chordata	77.3698559	8.28327852	1400 m
*Dendrobeania* sp. (A18)	Bryozoa	77.0797973	8.86340588	2200 m
*Tricellaria ternata* (A22)	Bryozoa	76.1558945	8.49651083	2200 m
*Chlamys islandica* (A23)	Mollusca	75.8646908	9.83376993	2300 m
*Halichondria panicea* (HP)	Porifera	69.6439	18.7369	Lower intertidal

## Data Availability

The 16S rRNA gene sequences from the culturable Actinobacteria isolates have been deposited in GenBank under accession numbers PQ814116-PQ814140. For metabarcoding data, demultiplexed, high-quality sequence reads have been deposited in the NCBI Sequence Read Archive (SRA) under BioProject ID: PRJNA1266703.

## Supplementary Materials

The following supporting information can be downloaded at: https://www.mdpi.com/article/10.3390/md23100408/s1, Figure S1: phylogenetic tree of culturable actinobacteria; Figure S2: Rarefaction curves of microbial diversity; Figure S3: Total number of Actinobacteria ASVs; Figure S4: Alpha diversity metrics of Actinobacteria communities; Figure S5: High-throughput co-cultivation assay; Figure S6: Co-culturing Morphological phenotype; Table S1: ASVs table; Table S2: ASVs_isolates Blast.

## References

[1] Louca S., Mazel F., Doebeli M., Parfrey L.W. (2019). A Census-Based Estimate of Earth’s Bacterial and Archaeal Diversity. PLoS Biol..

[2] Salwan R., Sharma V. (2020). Molecular and Biotechnological Aspects of Secondary Metabolites in Actinobacteria. Microbiol. Res..

[3] Robertsen H.L., Musiol-Kroll E.M. (2019). Actinomycete-Derived Polyketides as a Source of Antibiotics and Lead Structures for the Development of New Antimicrobial Drugs. Antibiotics.

[4] Macagnan D., Romeiro R.S., De Souza J.T., Pomella A.W.V. (2006). Isolation of Actinomycetes and Endospore-Forming Bacteria from the Cacao Pod Surface and Their Antagonistic Activity against the Witches’ Broom and Black Pod Pathogens. Phytoparasitica.

[5] Mayfield C.I., Williams S.T., Ruddick S.M., Hatfield H.L. (1972). Studies on the Ecology of Actinomycetes in Soil IV. Observations on the Form and Growth of Streptomycetes in Soil. Soil Biol. Biochem..

[6] Embley T.M., Stackebrandt E. (1994). The Molecular Phylogeny and Systematics of the Actinomycetes. Annu. Rev. Microbiol..

[7] Bérdy J. (2012). Thoughts and Facts about Antibiotics: Where We Are Now and Where We Are Heading. J. Antibiot..

[8] Barka E.A., Vatsa P., Sanchez L., Gaveau-Vaillant N., Jacquard C., Klenk H.-P., Clément C., Ouhdouch Y., van Wezel G.P. (2016). Taxonomy, Physiology, and Natural Products of Actinobacteria. Microbiol. Mol. Biol. Rev..

[9] Rotter A., Barbier M., Bertoni F., Bones A.M., Cancela M.L., Carlsson J., Carvalho M.F., Cegłowska M., Chirivella-Martorell J., Conk Dalay M. (2021). The Essentials of Marine Biotechnology. Front. Mar. Sci..

[10] Subramani R., Aalbersberg W. (2012). Marine Actinomycetes: An Ongoing Source of Novel Bioactive Metabolites. Microbiol. Res..

[11] Knobloch S., Jóhannsson R., Marteinsson V. (2019). Co-Cultivation of the Marine Sponge Halichondria Panicea and Its Associated Microorganisms. Sci. Rep..

[12] Rutledge P.J., Challis G.L. (2015). Discovery of Microbial Natural Products by Activation of Silent Biosynthetic Gene Clusters. Nat. Rev. Microbiol..

[13] Peng X.Y., Wu J.T., Shao C.L., Li Z.Y., Chen M., Wang C.Y. (2021). Co-Culture: Stimulate the Metabolic Potential and Explore the Molecular Diversity of Natural Products from Microorganisms. Mar. Life Sci. Technol..

[14] Kapoore R.V., Padmaperuma G., Maneein S., Vaidyanathan S. (2022). Co-Culturing Microbial Consortia: Approaches for Applications in Biomanufacturing and Bioprocessing. Crit. Rev. Biotechnol..

[15] Patricia G., Olaf T., Kirsten K., Martin T., van de Velde C., Swatantar K., Paolina G. (2018). Growth Promotion and Inhibition Induced by Interactions of Groundwater Bacteria. FEMS Microbiol. Ecol..

[16] Tyc O., van den Berg M., Gerards S., van Veen J.A., Raaijmakers J.M., de Boer W., Garbeva P. (2014). Impact of Interspecific Interactions on Antimicrobial Activity among Soil Bacteria. Front. Microbiol..

[17] Cole J.R., Chai B., Farris R.J., Wang Q., Kulam-Syed-Mohideen A.S., McGarrell D.M., Bandela A.M., Cardenas E., Garrity G.M., Tiedje J.M. (2007). The Ribosomal Database Project (RDP-II): Introducing MyRDP Space and Quality Controlled Public Data. Nucleic Acids Res..

[18] Kim M., Oh H.S., Park S.C., Chun J. (2014). Towards a Taxonomic Coherence between Average Nucleotide Identity and 16S RRNA Gene Sequence Similarity for Species Demarcation of Prokaryotes. Int. J. Syst. Evol. Microbiol..

[19] HAMEŞ-KOCABAŞ E.E., UZEL A. (2012). Isolation Strategies of Marine-Derived Actinomycetes from Sponge and Sediment Samples. J. Microbiol. Methods.

[20] Schneider Y.K., Hagestad O.C., Li C., Hansen E.H., Andersen J.H. (2022). Selective Isolation of Arctic Marine Actinobacteria and a Down-Scaled Fermentation and Extraction Strategy for Identifying Bioactive Compounds. Front. Microbiol..

[21] Hentschel U., Piel J., Degnan S.M., Taylor M.W. (2012). Genomic Insights into the Marine Sponge Microbiome. Nat. Rev. Microbiol..

[22] Zhang H., Zhang W., Jin Y., Jin M., Yu X. (2008). A Comparative Study on the Phylogenetic Diversity of Culturable Actinobacteria Isolated from Five Marine Sponge Species. Antonie Van Leeuwenhoek Int. J. General. Mol. Microbiol..

[23] Schneemann I., Nagel K., Kajahn I., Labes A., Wiese J., Imhoff J.F. (2010). Comprehensive Investigation of Marine Actinobacteria Associated with the Sponge Halichondria Panicea. Appl. Environ. Microbiol..

[24] Anteneh Y.S., Yang Q., Brown M.H., Franco C.M.M. (2022). Factors Affecting the Isolation and Diversity of Marine Sponge-Associated Bacteria. Appl. Microbiol. Biotechnol..

[25] Versluis D., McPherson K., van Passel M.W.J., Smidt H., Sipkema D. (2017). Recovery of Previously Uncultured Bacterial Genera from Three Mediterranean Sponges. Mar. Biotechnol..

[26] Miksch S., Meiners M., Meyerdierks A., Probandt D., Wegener G., Titschack J., Jensen M.A., Ellrott A., Amann R., Knittel K. (2021). Bacterial Communities in Temperate and Polar Coastal Sands Are Seasonally Stable. ISME Commun..

[27] Charalampous G., Kormas K.A., Antoniou E., Kalogerakis N., Gontikaki E. (2024). Distinct Communities of Bacteria and Unicellular Eukaryotes in the Different Water Masses of Cretan Passage Water Column (Eastern Mediterranean Sea). Curr. Microbiol..

[28] Reveillaud J., Maignien L., Eren M.A., Huber J.A., Apprill A., Sogin M.L., Vanreusel A. (2014). Host-Specificity among Abundant and Rare Taxa in the Sponge Microbiome. ISME J..

[29] Schmidt M.L., Biddanda B.A., Weinke A.D., Chiang E., Januska F., Props R., Denef V.J. (2020). Microhabitats Are Associated with Diversity-Productivity Relationships in Freshwater Bacterial Communities. FEMS Microbiol. Ecol..

[30] Scheuerl T., Hopkins M., Nowell R.W., Rivett D.W., Barraclough T.G., Bell T. (2020). Bacterial Adaptation Is Constrained in Complex Communities. Nat. Commun..

[31] Singh R.P., Manchanda G., Bhattacharjee K., Panosyan H. (2022). Microbes in Microbial Communities: Ecological and Applied Perspectives.

[32] Schwaha T., Hirose M., Wanninger A. (2016). The Life of the Freshwater Bryozoan Stephanella Hina (Bryozoa, Phylactolaemata)—A Crucial Key to Elucidating Bryozoan Evolution. Zool. Lett..

[33] Schreiber L., Kjeldsen K.U., Funch P., Jensen J., Obst M., López-Legentil S., Schramm A. (2016). Endozoicomonas Are Specific, Facultative Symbionts of Sea Squirts. Front. Microbiol..

[34] Chen L., Hu J.S., Xu J.L., Shao C.L., Wang G.Y. (2018). Biological and Chemical Diversity of Ascidian-Associated Microorganisms. Mar. Drugs.

[35] Tang H., Shi X., Wang X., Hao H., Zhang X.M., Zhang L.P. (2016). Environmental Controls over Actinobacteria Communities in Ecological Sensitive Yanshan Mountains Zone. Front. Microbiol..

[36] Zhang B., Wu X., Tai X., Sun L., Wu M., Zhang W., Chen X., Zhang G., Chen T., Liu G. (2019). Variation in Actinobacterial Community Composition and Potential Function in Different Soil Ecosystems Belonging to the Arid Heihe River Basin of Northwest China. Front. Microbiol..

[37] Chen L., Wang X.N., Fu C.M., Wang G.Y. (2019). Phylogenetic Analysis and Screening of Antimicrobial and Antiproliferative Activities of Culturable Bacteria Associated with the Ascidian Styela Clava from the Yellow Sea, China. Biomed. Res. Int..

[38] Kim S.H., Yang H.O., Sohn Y.C., Kwon H.C. (2010). Aeromicrobium Halocynthiae Sp. Nov., a Taurocholic Acid-Producing Bacterium Isolated from the Marine Ascidian Halocynthia Roretzi. Int. J. Syst. Evol. Microbiol..

[39] Johnson J.S., Spakowicz D.J., Hong B.Y., Petersen L.M., Demkowicz P., Chen L., Leopold S.R., Hanson B.M., Agresta H.O., Gerstein M. (2019). Evaluation of 16S RRNA Gene Sequencing for Species and Strain-Level Microbiome Analysis. Nat. Commun..

[40] Teng F., Darveekaran Nair S.S., Zhu P., Li S., Huang S., Li X., Xu J., Yang F. (2018). Impact of DNA Extraction Method and Targeted 16S-RRNA Hypervariable Region on Oral Microbiota Profiling. Sci. Rep..

[41] Carrigg C., Rice O., Kavanagh S., Collins G., O’Flaherty V. (2007). DNA Extraction Method Affects Microbial Community Profiles from Soils and Sediment. Appl. Microbiol. Biotechnol..

[42] Aßhauer K.P., Wemheuer B., Daniel R., Meinicke P. (2015). Tax4Fun: Predicting Functional Profiles from Metagenomic 16S RRNA Data. Bioinformatics.

[43] Wu J., Guan T., Jiang H., Zhi X., Tang S., Dong H., Zhang L., Li W. (2009). Diversity of Actinobacterial Community in Saline Sediments from Yunnan and Xinjiang, China. Extremophiles.

[44] Nichols D., Lewis K., Orjala J., Mo S., Ortenberg R., O’Connor P., Zhao C., Vouros P., Kaeberlein T., Epstein S.S. (2008). Short Peptide Induces an “Uncultivable” Microorganism to Grow in Vitro. Appl. Environ. Microbiol..

[45] Bao Y., Dolfing J., Guo Z., Chen R., Wu M., Li Z., Lin X., Feng Y. (2021). Important Ecophysiological Roles of Non-Dominant Actinobacteria in Plant Residue Decomposition, Especially in Less Fertile Soils. Microbiome.

[46] Větrovský T., Baldrian P. (2015). An In-Depth Analysis of Actinobacterial Communities Shows Their High Diversity in Grassland Soils along a Gradient of Mixed Heavy Metal Contamination. Biol. Fertil. Soils.

[47] Peter G.V., Victor S. (1996). Organism Life Cycles, Predation, and the Structure of Marine Pelagic Ecosystems. Mar. Ecol. Prog. Ser..

[48] Lima-Mendez G., Faust K., Henry N., Decelle J., Colin S., Carcillo F., Chaffron S., Cesar Ignacio-Espinosa J., Roux S., Vincent F. (2015). Determinants of Community Structure in the Global Plankton Interactome. Science.

[49] Hibbing M.E., Fuqua C., Parsek M.R., Peterson S.B. (2010). Bacterial Competition: Surviving and Thriving in the Microbial Jungle. Nat. Rev. Microbiol..

[50] Selegato D.M., Castro-Gamboa I. (2023). Enhancing Chemical and Biological Diversity by Co-Cultivation. Front. Microbiol..

[51] Abdelmohsen U.R., Bayer K., Hentschel U. (2014). Diversity, Abundance and Natural Products of Marine Sponge-Associated Actinomycetes. Nat. Prod. Rep..

[52] Marmann A., Aly A.H., Lin W., Wang B., Proksch P. (2014). Co-Cultivation—A Powerful Emerging Tool for Enhancing the Chemical Diversity of Microorganisms. Mar. Drugs.

[53] Wakefield J., Hassan H.M., Jaspars M., Ebel R., Rateb M.E. (2017). Dual Induction of New Microbial Secondary Metabolites by Fungal Bacterial Co-Cultivation. Front. Microbiol..

[54] Nadell C.D., Drescher K., Foster K.R. (2016). Spatial Structure, Cooperation and Competition in Biofilms. Nat. Rev. Microbiol..

[55] Fuqua W.C., Winans S.C., Peter Greenberg E. (1994). Quorum Sensing in Bacteria: The LuxR-LuxI Family of Cell Density-Responsive Transcriptional Regulatorst. J. Bacteriol..

[56] Peterson S.B., Bertolli S.K., Mougous J.D. (2020). The Central Role of Interbacterial Antagonism in Bacterial Life. Curr. Biol..

[57] Zhu H., Sandiford S.K., Van Wezel G.P. (2014). Triggers and Cues That Activate Antibiotic Production by Actinomycetes. J. Ind. Microbiol. Biotechnol..

[58] Kehe J., Kulesa A., Ortiz A., Ackerman C.M., Thakku S.G., Sellers D., Kuehn S., Gore J., Friedman J., Blainey P.C. (2019). Massively Parallel Screening of Synthetic Microbial Communities. Proc. Natl. Acad. Sci. USA.

[59] Law J.W.F., Ser H.L., Ab Mutalib N.S., Saokaew S., Duangjai A., Khan T.M., Chan K.G., Goh B.H., Lee L.H. (2019). Streptomyces Monashensis Sp. Nov., a Novel Mangrove Soil Actinobacterium from East Malaysia with Antioxidative Potential. Sci. Rep..

[60] Donald L., Pipite A., Subramani R., Owen J., Keyzers R.A., Taufa T. (2022). Streptomyces: Still the Biggest Producer of New Natural Secondary Metabolites, a Current Perspective. Microbiol. Res..

[61] Palkova L., Tomova A., Repiska G., Babinska K., Bokor B., Mikula I., Minarik G., Ostatnikova D., Soltys K. (2021). Evaluation of 16S RRNA Primer Sets for Characterisation of Microbiota in Paediatric Patients with Autism Spectrum Disorder. Sci. Rep..

[62] Kearse M., Moir R., Wilson A., Stones-Havas S., Cheung M., Sturrock S., Buxton S., Cooper A., Markowitz S., Duran C. (2012). Geneious Basic: An Integrated and Extendable Desktop Software Platform for the Organization and Analysis of Sequence Data. Bioinformatics.

[63] Altschup S.F., Gish W., Miller W., Myers E.W., Lipman D.J. (1990). Basic Local Alignment Search Tool. J. Mol. Biol..

[64] Katoh K., Standley D.M. (2013). MAFFT Multiple Sequence Alignment Software Version 7: Improvements in Performance and Usability. Mol. Biol. Evol..

[65] Nguyen L.T., Schmidt H.A., Von Haeseler A., Minh B.Q. (2015). IQ-TREE: A Fast and Effective Stochastic Algorithm for Estimating Maximum-Likelihood Phylogenies. Mol. Biol. Evol..

[66] Minh B.Q., Nguyen M.A.T., Von Haeseler A. (2013). Ultrafast Approximation for Phylogenetic Bootstrap. Mol. Biol. Evol..

[67] Guindon S., Dufayard J.F., Lefort V., Anisimova M., Hordijk W., Gascuel O. (2010). New Algorithms and Methods to Estimate Maximum-Likelihood Phylogenies: Assessing the Performance of PhyML 3.0. Syst. Biol..

[68] Letunic I., Bork P. (2021). Interactive Tree of Life (ITOL) v5: An Online Tool for Phylogenetic Tree Display and Annotation. Nucleic Acids Res..

[69] Parada A.E., Needham D.M., Fuhrman J.A. (2016). Every Base Matters: Assessing Small Subunit RRNA Primers for Marine Microbiomes with Mock Communities, Time Series and Global Field Samples. Environ. Microbiol..

[70] Bolger A.M., Lohse M., Usadel B. (2014). Trimmomatic: A Flexible Trimmer for Illumina Sequence Data. Bioinformatics.

[71] Ewels P., Magnusson M., Lundin S., Käller M. (2016). MultiQC: Summarize Analysis Results for Multiple Tools and Samples in a Single Report. Bioinformatics.

[72] Bolyen E., Guerrini C.J., Botkin J.R., McGuire A.L. (2019). Reproducible, Interactive, Scalable and Extensible Microbiome Data Science Using QIIME 2. Nat. Biotechnol..

[73] Callahan B.J., McMurdie P.J., Rosen M.J., Han A.W., Johnson A.J.A., Holmes S.P. (2016). DADA_2_: High-Resolution Sample Inference from Illumina Amplicon Data. Nat. Methods.

[74] Rognes T., Flouri T., Nichols B., Quince C., Mahé F. (2016). VSEARCH: A Versatile Open Source Tool for Metagenomics. PeerJ.

[75] Quast C., Pruesse E., Yilmaz P., Gerken J., Schweer T., Yarza P., Peplies J., Glöckner F.O. (2013). The SILVA Ribosomal RNA Gene Database Project: Improved Data Processing and Web-Based Tools. Nucleic Acids Res..

[76] Oksanen J., Blanchet F.G., Kindt R., Legendre P., Minchin P.R., O’Hara R.B., Simpson G.L., Solymos P., Stevens M.H.H., Wagner H. Vegan: Community Ecology Package 2013. https://vegandevs.github.io/vegan/.

[77] McMurdie P.J., Holmes S. (2013). Phyloseq: An R Package for Reproducible Interactive Analysis and Graphics of Microbiome Census Data. PLoS ONE.

[78] Chen H., Boutros P.C. (2011). VennDiagram: A Package for the Generation of Highly-Customizable Venn and Euler Diagrams in R. BMC Bioinform..

[79] Wickham H., Averick M., Bryan J., Chang W., McGowan L., François R., Grolemund G., Hayes A., Henry L., Hester J. (2019). Welcome to the Tidyverse. J. Open Source Softw..

[80] Minh B.Q., Schmidt H.A., Chernomor O., Schrempf D., Woodhams M.D., Von Haeseler A., Lanfear R., Teeling E. (2020). IQ-TREE 2: New Models and Efficient Methods for Phylogenetic Inference in the Genomic Era. Mol. Biol. Evol..

