# Supplementary material for: Biodiversity and Biological Interactions of Actinobacteria Associated with Deep Sea and Intertidal Marine Invertebrates
Source: Mar Drugs. 2025 Oct 17;23(10):408. doi: 10.3390/md23100408 (PMC12565852; doi:10.3390/md23100408)
Supplement: Supplementary file 1 [file marinedrugs-23-00408-s001.zip › SUPPLEMENTARY/Figure S2- Rarefaction curves of microbial diversity.pptx]

## Slide 1
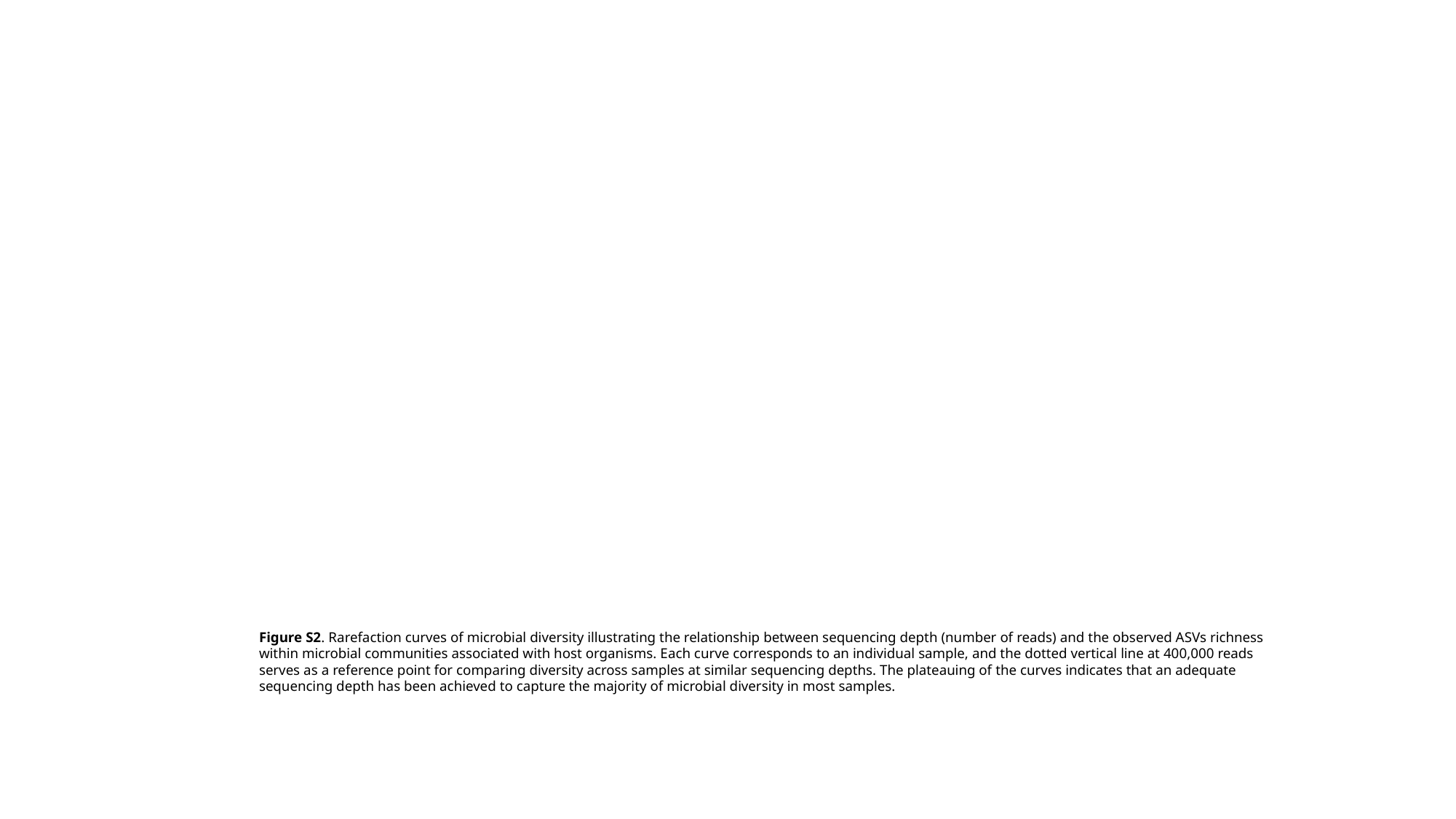

Figure S2. Rarefaction curves of microbial diversity illustrating the relationship between sequencing depth (number of reads) and the observed ASVs richness within microbial communities associated with host organisms. Each curve corresponds to an individual sample, and the dotted vertical line at 400,000 reads serves as a reference point for comparing diversity across samples at similar sequencing depths. The plateauing of the curves indicates that an adequate sequencing depth has been achieved to capture the majority of microbial diversity in most samples.
