# Supplementary material for: Biodiversity and Biological Interactions of Actinobacteria Associated with Deep Sea and Intertidal Marine Invertebrates
Source: Mar Drugs. 2025 Oct 17;23(10):408. doi: 10.3390/md23100408 (PMC12565852; doi:10.3390/md23100408)
Supplement: Supplementary file 1 [file marinedrugs-23-00408-s001.zip › SUPPLEMENTARY/Figure S3- Total number of Actinobacteria ASVs .pptx]

## Slide 1
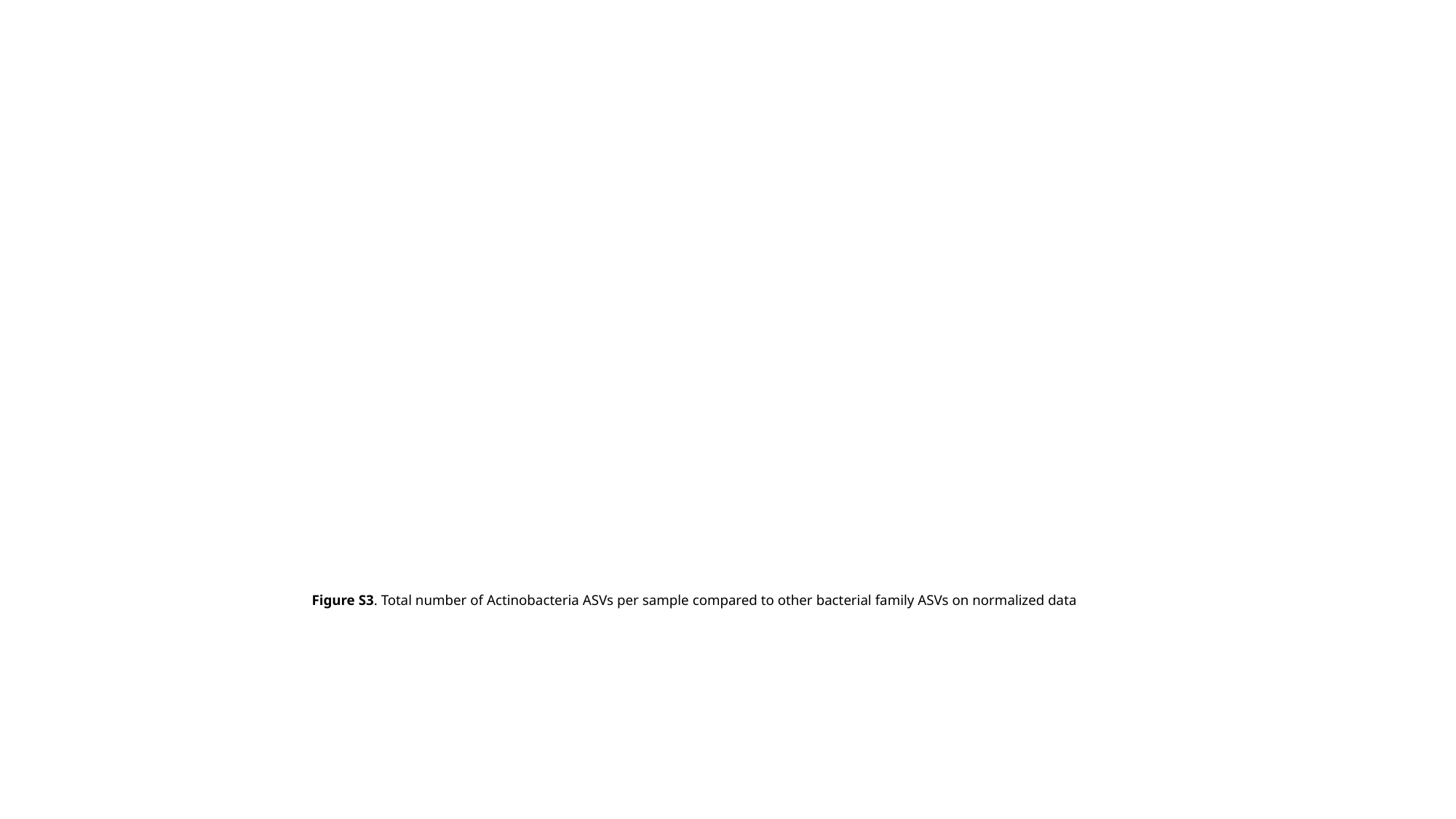

Figure S3. Total number of Actinobacteria ASVs per sample compared to other bacterial family ASVs on normalized data
