# Supplementary material for: Biodiversity and Biological Interactions of Actinobacteria Associated with Deep Sea and Intertidal Marine Invertebrates
Source: Mar Drugs. 2025 Oct 17;23(10):408. doi: 10.3390/md23100408 (PMC12565852; doi:10.3390/md23100408)
Supplement: Supplementary file 1 [file marinedrugs-23-00408-s001.zip › SUPPLEMENTARY/Figure S4- Alpha diversity metrics of Actinobacteria communities.pptx]

## Slide 1
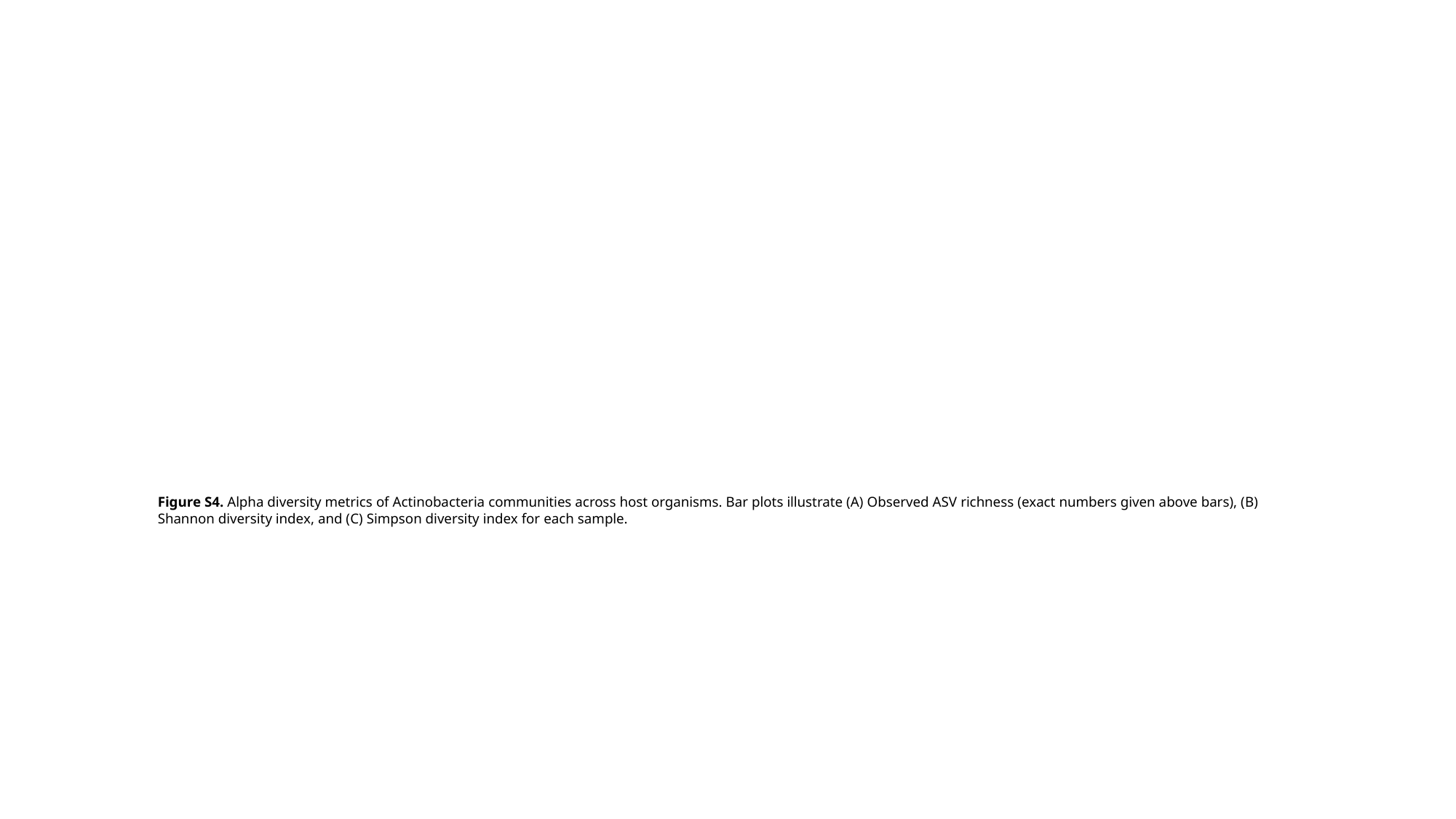

Figure S4. Alpha diversity metrics of Actinobacteria communities across host organisms. Bar plots illustrate (A) Observed ASV richness (exact numbers given above bars), (B) Shannon diversity index, and (C) Simpson diversity index for each sample.
