# Supplementary material for: Biodiversity and Biological Interactions of Actinobacteria Associated with Deep Sea and Intertidal Marine Invertebrates
Source: Mar Drugs. 2025 Oct 17;23(10):408. doi: 10.3390/md23100408 (PMC12565852; doi:10.3390/md23100408)
Supplement: Supplementary file 1 [file marinedrugs-23-00408-s001.zip › SUPPLEMENTARY/Figure S5- High-throughput co-cultivation assay.pptx]

## Slide 1
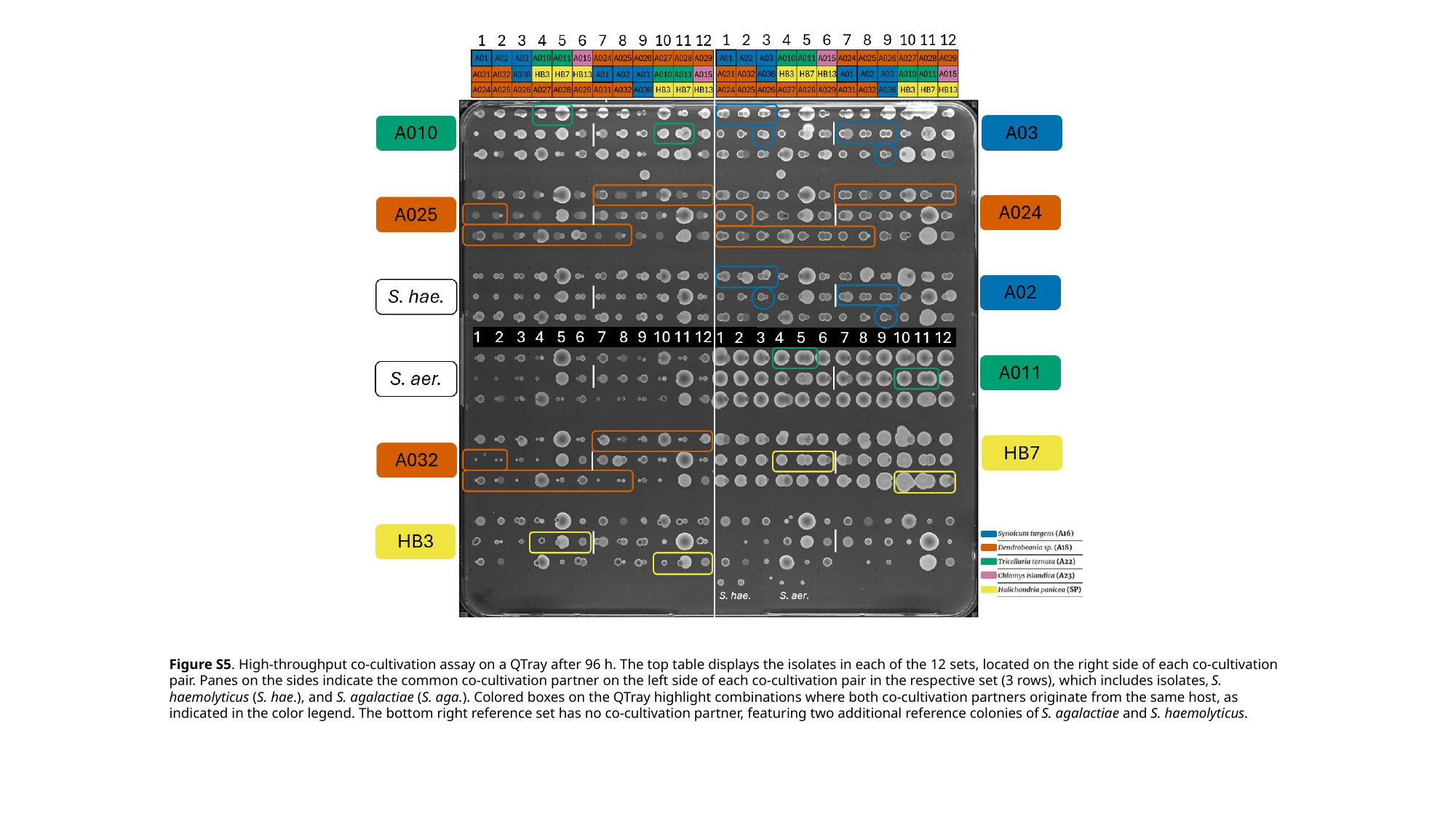

Figure S5. High-throughput co-cultivation assay on a QTray after 96 h. The top table displays the isolates in each of the 12 sets, located on the right side of each co-cultivation pair. Panes on the sides indicate the common co-cultivation partner on the left side of each co-cultivation pair in the respective set (3 rows), which includes isolates, S. haemolyticus (S. hae.), and S. agalactiae (S. aga.). Colored boxes on the QTray highlight combinations where both co-cultivation partners originate from the same host, as indicated in the color legend. The bottom right reference set has no co-cultivation partner, featuring two additional reference colonies of S. agalactiae and S. haemolyticus.
