# Supplementary material for: Biodiversity and Biological Interactions of Actinobacteria Associated with Deep Sea and Intertidal Marine Invertebrates
Source: Mar Drugs. 2025 Oct 17;23(10):408. doi: 10.3390/md23100408 (PMC12565852; doi:10.3390/md23100408)
Supplement: Supplementary file 1 [file marinedrugs-23-00408-s001.zip › SUPPLEMENTARY/Figure S6- Co-culturing Morphological phenotype.pptx]

## Slide 1
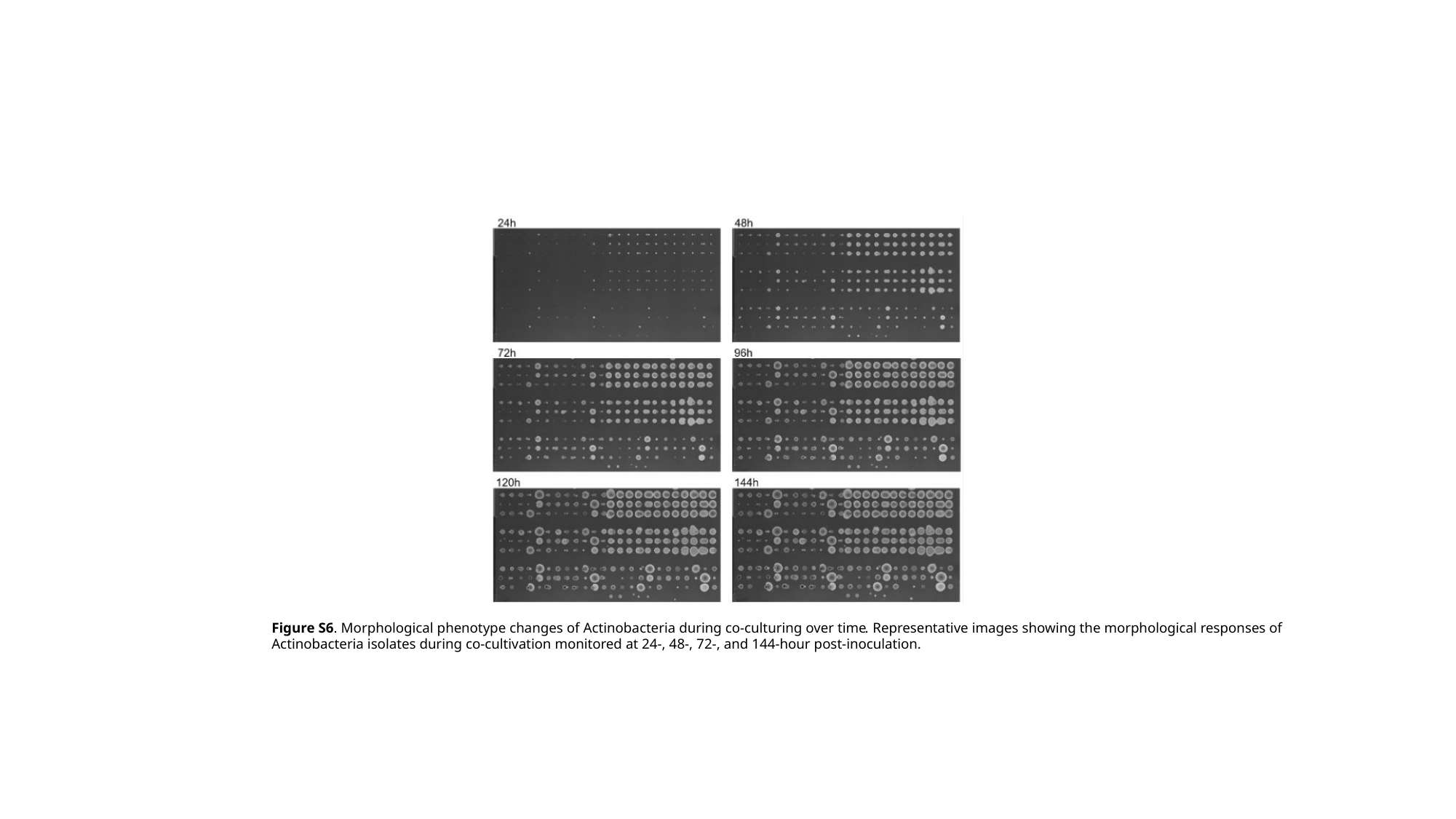

Figure S6. Morphological phenotype changes of Actinobacteria during co-culturing over time. Representative images showing the morphological responses of Actinobacteria isolates during co-cultivation monitored at 24-, 48-, 72-, and 144-hour post-inoculation.
